# Quantifying the impacts of diverse vegetation-covered patterns on hillslope soil erosion: a case experiment of alfalfa-covered hillslopes

**DOI:** 10.3389/fpls.2025.1629542

**Published:** 2025-08-25

**Authors:** Chong Yao, Qingwei Zhang, Kebing Chen, Shige Zhang, Ming Zhu, Zhijia Gu, Wei Yan, Faqi Wu

**Affiliations:** ^1^ North-South Transitional Zone Typical Vegetation Phenology Observation and Research Station of Henan Province, School of Geographic Sciences, Xinyang Normal University, Xinyang, Henan, China; ^2^ Country College of Soil and Water Conservation Science and Engineering, Northwest A&F University, Xianyang, Shaanxi, China; ^3^ China Railway Siyuan Survey and Design Group Co., Ltd., Wuhan, Hubei, China

**Keywords:** soil erosion, alfalfa-cover hillslopes, rainfall simulation, RRB and SRB, the Loess Plateau

## Abstract

**Introduction:**

The discrepancies in near-soil-surface hydrologic processes triggered by herbage spatial distribution pattern greatly influence the variation in hillslope erosion process. However, knowledge about the influence of herbage spatial distribution pattern on hillslope erosion is still limited.

**Methods:**

In the current study, runoff plots (length × width × depth, 2 × 1 × 0.5 m) with slope gradient of 8.75%–36.40% and a side-spray rainfall simulator with rainfall intensity of 90 mm h^-1^ were adopted to rainfall simulation. Four herbage spatial distribution patterns with vegetation coverage of 50% (US, upper hillslopes; MS, middle hillslopes; LS, downstream hillslopes; and SS, equally spaced planting) and bare soil (CK) were arranged to quantify the response of hillslope erosion process to alfalfa spatial distribution pattern.

**Results:**

The results indicated that the initial runoff generation time followed the order of CK, US, MS, LS, and SS. Compared with CK, the mean runoff rate and sediment yield rate for alfalfa-covered hillslopes decreased by 7.18% to 83.77% and 12.62% to 85.69%, and sediment concentration decreased by 0.26 to 2.22 g L^-1^. The sediment reduction benefits (SRB) and runoff reduction benefits (RRB) followed the order of SS, LS, MS, and US. The average infiltration rates for CK and alfalfa-covered hillslopes with slope gradient of 8.75%–36.40% were 0.17 to 0.50 and 0.28 to 1.35 mm m^-2^ min^-1^, respectively.

**Discussion:**

As the results of shielding and protection effect of alfalfa, initial runoff generation time was delayed, infiltration rate was enhanced, and runoff and sediment yield rates were reduced; thus, soil loss was lowered on alfalfa-covered hillslopes. The research provided scientific reference for understanding the anti-erosion of herbage spatial distribution pattern and theoretical guidance for formulating soil and water conservation planning.

## 1 Introduction

The frequent occurrence of soil erosion has severely constrained improvements in the ecological environment, human settlements, and high-quality development (Liu et al., 2020; Montgomery, 2007; Quinton et al., 2010). Natural factors such as loose soil and uneven precipitation, coupled with intense human activities, have made the Loess Plateau a significant source of soil erosion in China (Liu et al., 2020; Zhu et al., 2024). The implementation of the Grain for Green Program has significantly increased the vegetation cover in the Loess Plateau and improved the local ecological environment (Huang et al., 2023; Yang et al., 2025; Yibin et al., 2024). However, the regional environment carrying capacity is limited, and finite water and soil resources are insufficient to support vegetation cover across the entire area, particularly in arid and semi-arid regions (Chen et al., 2024b; Feng et al., 2016). Selecting appropriate vegetation species and establishing rational and efficient vegetation cover pattern are crucial to enhance the ecological functioning of vegetation in the Loess Plateau. Therefore, research on the relationship between vegetation spatial distribution patterns and soil erosion in fragile ecological regions has become a prominent topic and a critical scientific issue that urgently requires resolution.

Vegetation cover has demonstrated good erosion prevention function through the combined effects of canopy interception, litter water storage, and rainfall energy dissipation (Hu et al., 2023; Van Dijk and Bruijnzeel, 2001; Yubonchit et al., 2025). As a result of the excellent erosion prevention provided by grassland vegetation, soil erosion rate in grasslands has been found to be lower than those in croplands (Li et al., 2024b; Zhang et al., 2024a). The vegetation canopy mitigates the effect of rainfall impact through rainfall redistribution and its shielding (Niu et al., 2024; Wang et al., 2024b; Zhang et al., 2024b). Vegetation cover is a critical indicator affecting hillslope erosion, with studies showing that 50% of vegetation cover could effectively decrease soil and water loss (Zi et al., 2024). Zheng et al. (2021) found that grass cover could reduce sediment by 97.84% to 98.81% and runoff by 47.28% to 82.12%. Vegetation cover could reduce hillslope soil erosion by prolonging the initial runoff time and increasing infiltration (Lin et al., 2019).

Differences in vegetation spatial distribution patterns may exert varying regulatory effects on runoff and on sediment (Entezami et al., 2024; Zi et al., 2024). Vegetation spatial distribution pattern refers to the spatial configuration of vegetation with varying quantities, sizes, and types within a region. Vegetation pattern influences the water–sediment relationship on hillslopes with varying degrees (Dupuy et al., 2012; Puigdefábregas, 2005; Zheng et al., 2022). Vegetation patterns regulate the hillslope erosion process by affecting hydrological connectivity and the sediment transport capacity (Hu et al., 2023; Li et al., 2024b). Patch-scale observational studies have found that a smaller plot has greater hydrologic disconnection and lower runoff energy (Boix-Fayos et al., 2007). An increase in vegetation pattern coarseness has been associated with higher hillslope runoff and sediment yield, while greater vegetation patch density tends to reduce the runoff. Aggregated vegetation patches demonstrate a stronger capacity to retain soil particles as the degree of aggregation increases (Bautista et al., 2007). Zhang et al. (2014) found that *Artemisia capillaris* with distribution patterns of chessboard, strips, and long strips could effectively reduce soil erosion. Feng et al. (2018) conducted rainfall experiments with six planting patterns and found that adjusting the planting position of vegetation could effectively reduce water erosion. Although some research investigated erosion prevention for varying vegetation patterns, most have focused on total soil erosion, with limited attention given to the dynamic process involved. Therefore, further research is needed to explore the interactions between vegetation spatial patterns and hillslope erosion process.

Vegetation is a typically distributed patch form within bare or sparse areas (Bautista et al., 2007; Feng et al., 2018). The vegetation spatial distribution pattern regulates soil erosion by influencing soil properties and hydrological processes (Levia and Frost, 2003; Puigdefábregas, 2005; Wang et al., 2024a). Notable differences exist between bare land and vegetation-covered areas in terms of soil properties. Root exudates and interpenetration and fixation of vegetation roots improve soil organic matter, aggregate stability, and permeability (Ghestem et al., 2014; Nardi et al., 2002; Niu et al., 2024; Puigdefábregas, 2005). Plant roots supply necessary nutrients for growth and contribute significantly to soil development and improving the soil anti-erosion ability (De Baets et al., 2006; Nardi et al., 2000, 2002; Yang et al., 2024). Loades et al. (2010) conducted shear tests on barley root soil with different planting densities and indicated that soil shear strength with barley root increased by an average of 53%. Changes in soil properties, when covered by vegetation patches, result in lower runoff and sediment yields than in bare areas (Li et al., 2024b; Zhang et al., 2019). In addition, hydrological processes such as re-infiltration and sediment interception must be considered (Futerman et al., 2025; Puigdefábregas, 2005; Zhang et al., 2014). Vegetation patterns with high patch density intercept surface flow and increase the chance of re-infiltration (Leung et al., 2017; Vinatier et al., 2024). In contrast, the extent of bare soil is larger under low patch density, physical crust formation is more likely, and hydrological connectivity is enhanced, thereby reducing infiltration (Li et al., 2024b; Niu et al., 2024; Wang et al., 2022b). Without the shielding and protection of vegetation, surface runoff tends to have higher velocity, resulting in higher sediment concentrations (Futerman et al., 2025; Hu et al., 2023; Lin et al., 2025; Zhang et al., 2024b).

Soil erosion on the Loess Plateau is characterized by diverse erosion types and uneven temporal and spatial distribution (Liu et al., 2020; Wang et al., 2014). The effects of land use, field crops, agriculture practice, and vegetation restoration on hillslope erosion in arid and semi-arid areas have been widely investigated (Feng et al., 2016; Lin et al., 2025; Quinton et al., 2010; Zhang et al., 2019, 2024b). Alfalfa, a typical forage crop on the Loess Plateau, exhibits strong adaptability, rapid growth, high biomass, and strong nitrogen-fixing ability. It has been shown to effectively control soil erosion and improve soil fertility, offering good economic and ecological benefits (Xiao et al., 2015; Yao et al., 2023a). However, the effect of alfalfa on soil erosion process on varying slopes has rarely been quantified, and the effect of alfalfa with different spatial distribution patterns on variation in hillslope erosion process remains poorly understood. Therefore, the aims of this study were to (1) explore the variation in soil erosion process with different spatial alfalfa distribution patterns, (2) quantify the RRB and SRB on hillslopes planted with alfalfa, and (3) analyze the causes of differences in soil erosion among different alfalfa spatial distribution patterns.

## 2 Materials and methods

### 2.1 Study area

The Loess Plateau (33°41′–41°16′ N, 100°52′–114°33′ E) is located in northwest China, approximately 640,000 km^2^. The terrain of the Loess Plateau is dominated by hill and gully landscapes, with slope gradients mostly ranging from 24.9% to 53.2% (Lin et al., 2019). The Loess Plateau has a warm temperate continental monsoon climate, with precipitation and temperature decreasing from southeast to northwest. The annual average temperature ranges from 6°C to 14°C, and the annual average precipitation is 200–700 mm, with most rainfall occurring from July to September (Yao et al., 2023a). The soil is characterized by fine particles, soft texture, and abundant mineral nutrients, making it conducive to farming. However, its porous, loose, and poor structure, developed vertical joints, and high water permeability, combined with frequent summer rainstorms, have led to severe soil erosion. The test soil is collected from a local abandoned farmland with clay, silt, sand, and soil organic matter contents of 28.2%, 41.6%, 30.2%, and 19.17 g kg^–1^, respectively.

### 2.2 Rainfall device

**Figure 1:**
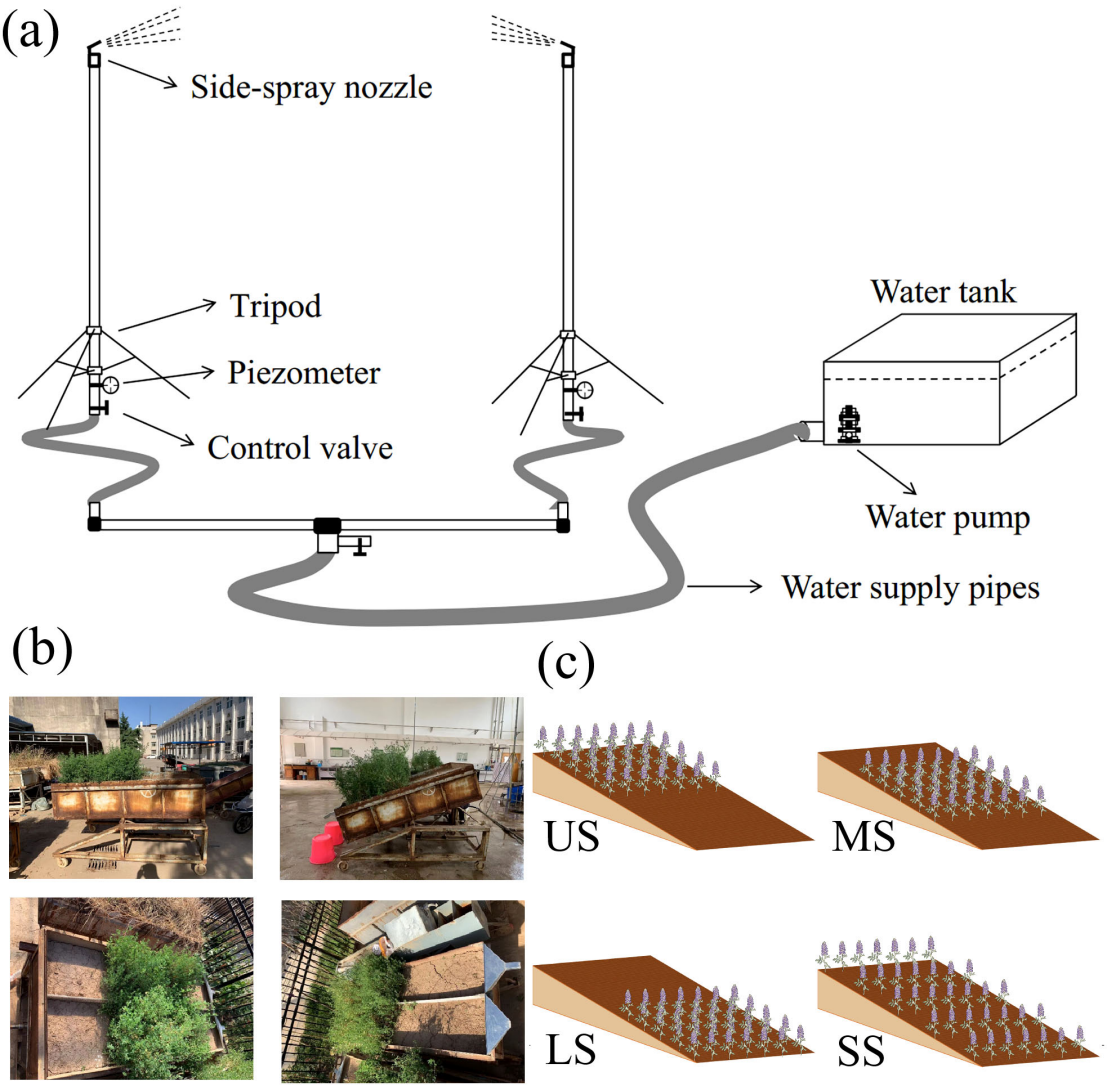
Side-spray rainfall simulator **(a)**, runoff plots **(b)**, and diagram of vegetation spatial distribution pattern **(c)**.

A side-spray rainfall simulator was used in the current experiment, developed by the Institute of Soil and Water Conservation, Chinese Academy of Sciences (**Figure 1a**), which has been widely used for rainfall simulation. The rainfall system consisted of two side-spray nozzles (5.0–12.0 mm), two brackets (7.5 m), two pressure gauges (0.10–0.15 MPa), a water pump (2.2 kW), a water tank (length of 4.0 m, width of 2.5 m, and depth of 2.5 m), and a water supply pipe (inner diameter of 48 mm). The nozzles were installed at the top of the brackets with a spray height of 1 m. The rainfall height of the rainfall simulator is 7.5 m, ensuring that raindrops reached near-terminal velocity. The effective rainfall area is 7 m × 5 m, with a rainfall uniformity of over 85%. The rainfall intensity with a range of 30–140 mm h^–1^ and raindrop diameter of 0.17–3.61 mm can be adjusted by regulating the water pressure control valve and replacing the nozzle gaskets. Before rainfall simulation, the rainfall intensity was calibrated to meet the experiment’s requirements.

### 2.3 Experiment designs

Rainfall simulation experiments were conducted at the Hydraulic Engineering Laboratory of Northwest A&F University, Shaanxi, China. Soil boxes (length of 2.0 m, width of 1.0 m, and depth of 0.5 m) were used for alfalfa planting and rainfall simulation. The test soil was collected from the top 0–20 cm of the arable layer of a local farmland in Yangling. The collected soil was air-dried and screened by using a 10-mm screen to eliminate the effect of root and other impurities. A 4-cm-thick layer of fine sand was placed at the bottom of the soil box to simulate natural drainage conditions. A porous jute sheet was used to separate the sand and soil layers. The soil was filled into the boxes in layers to achieve a bulk density of 1.25 g cm^-3^, based on field measurements in the local farmland. To ensure continuity between layers, each layer was gently scraped before adding the next. After filling, the soil was left under natural conditions for 30 days to simulate a natural soil state.

The experimental grass species was annual alfalfa, a typical forage on the Loess Plateau, which was sown at the end of April 2022. Soil boxes not planted with alfalfa were designed as bare soil control (CK). To explore the impact of vegetation spatial distribution patterns on hillslope erosion, four different vegetation pattern types with 50% vegetation cover were established: planting at the downstream hillslopes (LS), at the middle hillslopes (MS), and at the upper hillslopes (US) and equally spaced planting (SS) (**Figure 1b**). To ensure the hillslope vegetation coverage and different spatial alfalfa distribution patterns, the prepared soil boxes were divided into several soil plots (length × width, 0.2 m × 0.2 m). The grass seeds were evenly sown at specific positions on the hillslopes, and then the grass seeds were evenly mixed with the topsoil. Rainfall simulation experiments were conducted at the alfalfa podding stage. The planting area in all four vegetation spatial distribution patterns was kept the same, accounting for 50% of the entire hillslope area, ensuring consistent alfalfa coverage across all treatments. According to landform types of the Loess Plateau, the experimental runoff plots were established with four slope gradients of 8.75%, 17.63%, 26.80%, and 36.40% (Lin et al., 2019). Based on the long-term rainfall characteristics of the study area, a rainfall intensity of 90 mm h^–1^ was applied, with a simulation duration of 60 min.

### 2.4 Measurement of soil erosion indicator

After adjusting the rainfall intensity and slope gradient, the rainfall simulation experiment was initiated, and the soil erosion process was observed and recorded. The initial time to runoff was recorded, and the sediment samples were collected every 3 min using plastic buckets. The collected sediment samples were weighed, left to settle for 24 h, the supernatant was dropped, and then the sediment was dried at 105°C for 24 h before recording the dry weight. The runoff volume was calculated as the total mass of sediment and runoff minus the dry mass of sediment. Assuming constant rainfall intensity throughout the experiment, infiltration was estimated as the difference between total precipitation and runoff. In this study, the variables used to characterize the soil erosion process are initial time to runoff (*TR*), runoff rate (*RR*), sediment yield rate (*SR*), and infiltration rate (*RR*). These variables were calculated as follows (Equations 1–3):


(1) $$SR=\frac{Se}{st}$$

(2) $$RR=\frac{Rn}{st}=\frac{\left(ST-Se\right)}{1000\times st}$$

(3) $$IR=\frac{IT}{ts}=\frac{RT-Ri}{st}$$

where *SR*, *Se*, *s*, *t*, *RR*, *Rn*, *ST*, *IR*, *IT*, *RT*, and *Ri* are the sediment yield rate (g m^–2^ min^–1^), dry mass of collected sediment (g), runoff plot area (m^2^), sampling time (min), runoff rate (L m^–2^ min^–1^), runoff volume (L), mass of the collected sediment samples (g), infiltration rate (mm m^–2^ min^–1^), total infiltration amount (mm), total precipitation (mm), and runoff depth (mm), respectively. The runoff and sediment reduction benefits for alfalfa-covered hillslopes were calculated as follows (Equations 4–5):


(4) $$RRB=\frac{R_{ck-}R_{i}}{R_{ck}}\times 100\%$$

(5) $$SRB=\frac{S_{ck}-S_{i}}{S_{ck}}\times 100\%$$

where *RRB*, *R_ck_*, *R_i_*, *SRB*, *S_ck_*, and *S_i_* are the runoff reduction benefits, runoff volume for CK, runoff volume for alfalfa-covered hillslopes with different spatial distribution patterns, sediment reduction benefits, sediment yield for CK, and sediment yield for alfalfa-covered hillslopes with different spatial distribution patterns, respectively.

## 3 Results

### 3.1 The variability of initial runoff generation time

**Figure 2:**
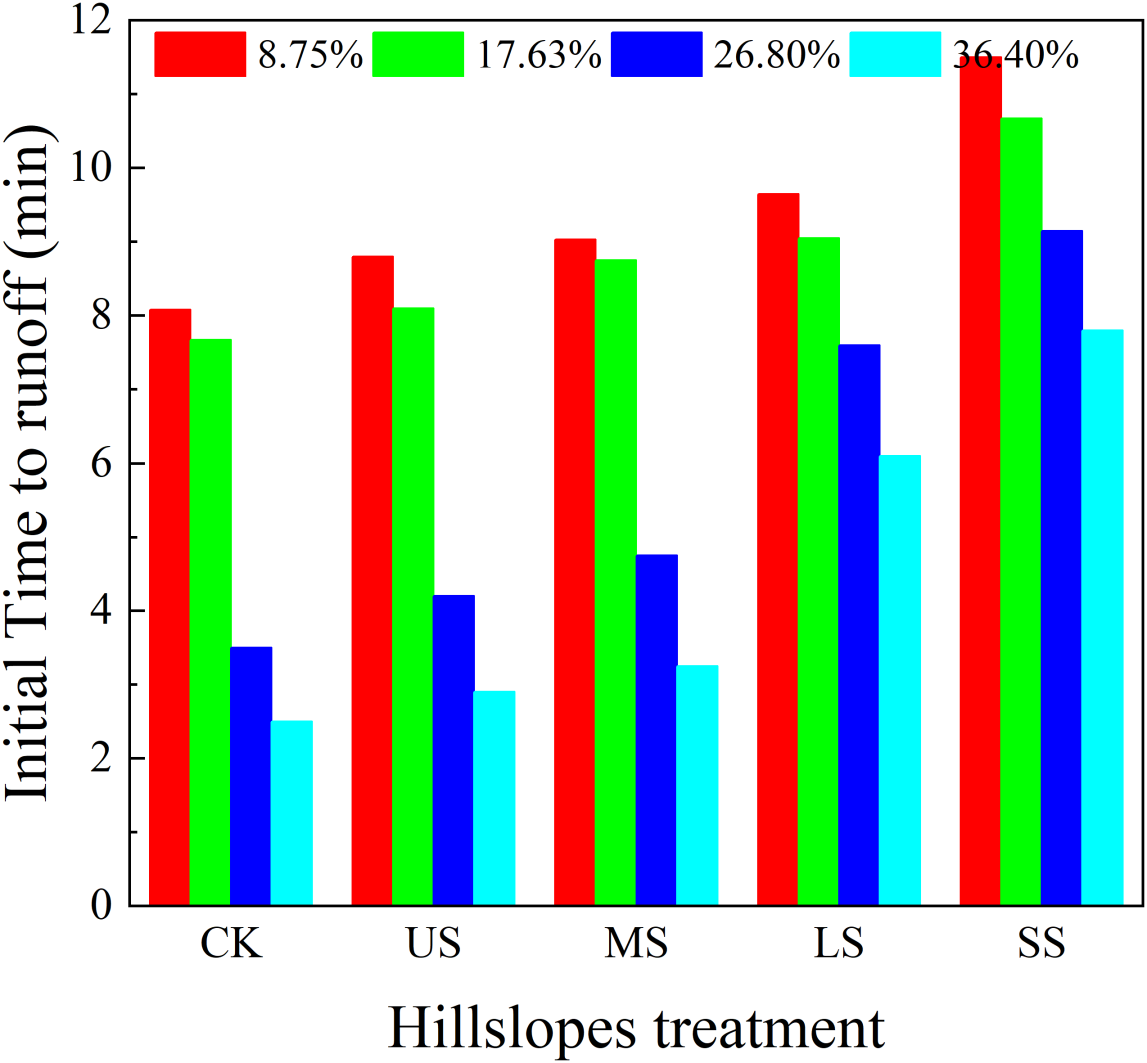
Variation in initial time to runoff with hillslope treatment and slope gradients.

The variation in initial runoff generation time for CK and alfalfa-covered hillslopes is shown in **Figure 2**. On the slope gradient of 8.75%–36.40%, the initial runoff generation time for CK and alfalfa-covered hillslopes with US, MS, LS, and SS pattern was 2.50 to 8.08, 2.90 to 8.80, 3.25 to 9.03, 6.10 to 9.65, and 7.80 to 11.50 min, respectively. The initial runoff generation time for the US, MS, LS, and SS alfalfa-covered hillslopes was delayed in comparison to CK. The initial runoff generation time with slope gradient of 17.63% to 36.40% for CK US, MS, LS, and SS patterns was advanced at 1.00 to 5.58, 1.30 to 5.90, 1.50 to 5.78, 1.50 to 3.55, and 1.35 to 3.70 min compared with those for the slope gradient of 8.75%, respectively, indicating that the initial runoff generation time was advanced with increasing slope gradient.

### 3.2 The variation in soil erosion process

#### 3.2.1 Runoff rate

**Figure 3:**
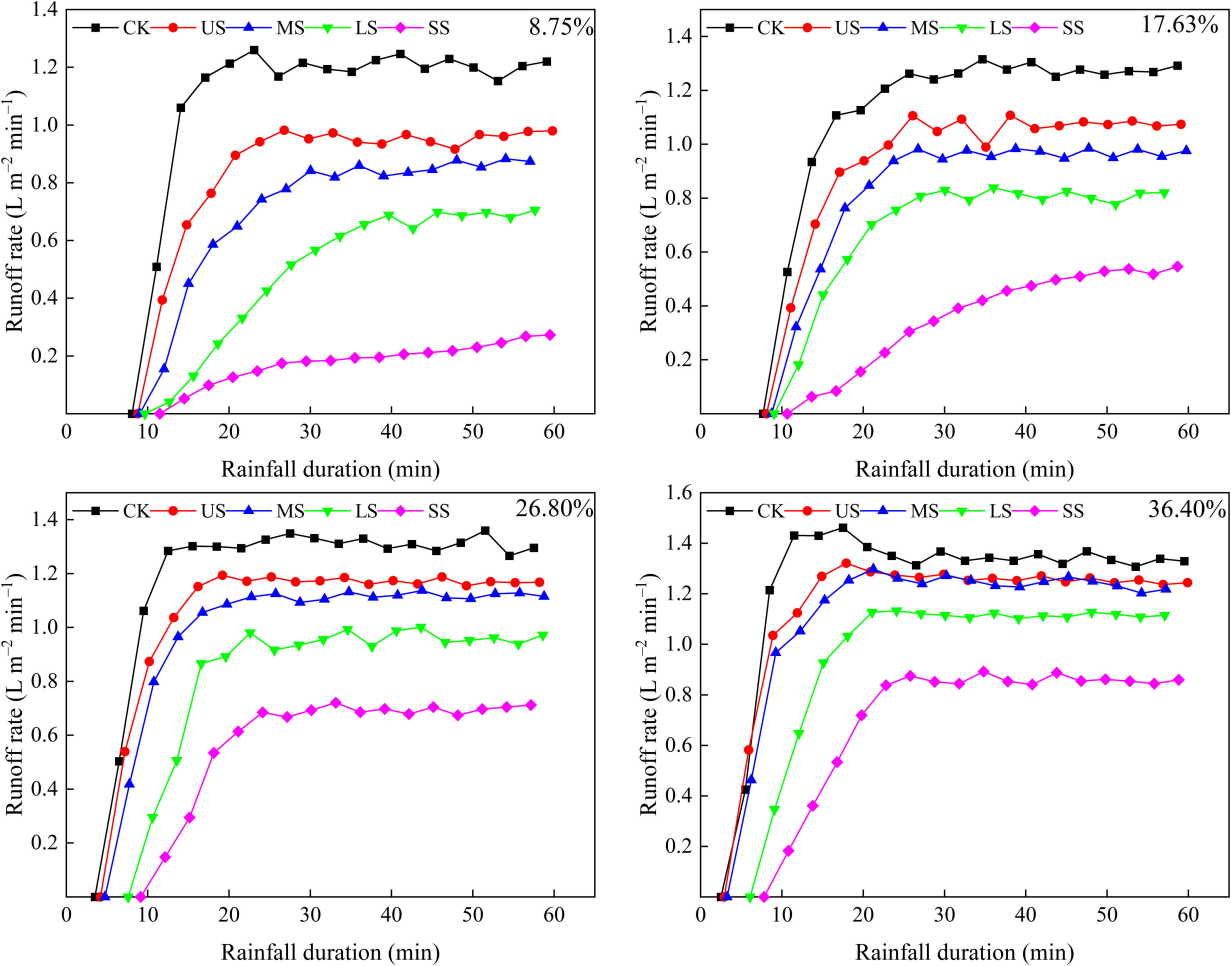
Variation in runoff rate with rainfall duration and slope gradient of 8.75%, 17.63%, 26.80%, and 36.40%.

The runoff rates for CK and alfalfa-covered hillslopes are shown in **Figure 3**, although the runoff rate on SS alfalfa-covered hillslopes with slope gradient of 8.75% and 17.63% showed a gradual increase without reaching a stable state during the entire rainfall. Overall, the runoff rate increased rapidly at first and was followed by stable fluctuations. For CK and alfalfa-covered hillslopes with slope gradient of 8.75%, 17.63%, 26.80%, and 36.40%, it took 17.08–39.65, 16.67–30.05, 12.50–24.15, and 11.50–22.80 min from the rainfall beginning to stable fluctuations in the runoff rate, respectively. The results indicated that the increase in slope gradient led to a more rapid transition from the initial increase in runoff rate to a stable state. While alfalfa cover delayed this transition, the SS pattern had the most significant delay effect.

Additionally, the average runoff rate was clearly affected by the slope gradient and vegetation spatial distribution patterns. At a slope gradient of 8.75%, the average runoff rates for CK, US, MS, LS, and SS were 1.09, 0.84, 0.71, 0.48, and 0.18 L m^–2^ min^–1^, respectively. At a slope gradient of 36.40%, the corresponding values were 1.24, 1.15, 1.11, 0.97, and 0.72 L m^–2^ min^–1^, respectively. Compared with CK, the average runoff rate for alfalfa-covered hillslopes at slope gradients of 8.75%, 17.63%, 26.80%, and 36.40% decreased by 22.91% to 83.77%, 16.85% to 69.27%, 11.06% to 50.79%, and 7.18% to 41.82%, respectively. These changes indicated that alfalfa cover could weaken the runoff rate, but the weakening effect decreased with slope gradient and differed with alfalfa spatial distribution patterns, with the SS pattern showing the best weakening effect.

#### 3.2.2 Sediment yield rate

**Figure 4:**
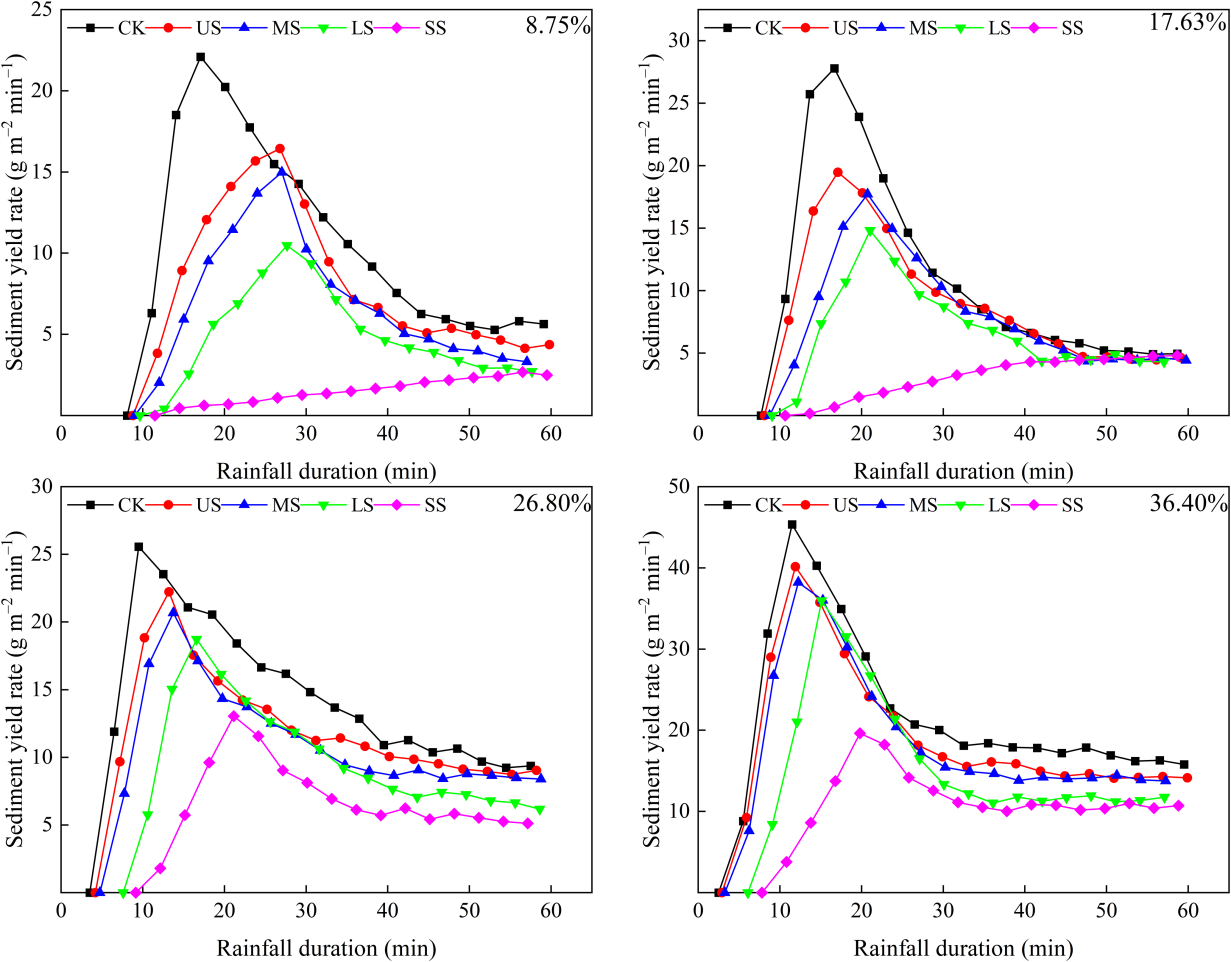
Variation in sediment yield rate with rainfall duration and slope gradient of 8.75%, 17.63%, 26.80%, and 36.40%.

The variability of sediment yield rate with rainfall duration for CK and alfalfa-covered plots is illustrated in **Figure 4**. Generally, the sediment yield rate increased rapidly with rainfall duration until reaching a peak, followed by a gradual decline. However, in the SS alfalfa-covered plots with slope gradients of 8.75% and 17.63%, the sediment yield rate gradually increased, without reaching a peak throughout the rainfall period. CK and alfalfa-covered hillslopes with slope gradients of 8.75%, 17.63%, 26.80%, and 36.40% took 17.08–27.65, 16.67–21.05, 9.50–21.15, and 11.50–19.80 min from the rainfall beginning to reach the peak sediment yield rates, respectively. These results indicated that increasing the slope gradient accelerated the attainment of peak sediment yield rate, but alfalfa coverage delayed this process.

Furthermore, the average and peak sediment yield rates showed obvious variations among different alfalfa spatial distribution patterns. The average sediment yield rate for CK and alfalfa-covered hillslopes with slope gradients of 8.75%, 17.63%, 26.80%, and 36.40% ranged from 1.50 to 10.4, 3.05 to 10.91, 6.53 to 14.04, and 10.93 to 21.32 g m^–2^ min^–1^, respectively. The corresponding peak sediment yield rates ranged from 10.74 to 22.09, 14.82 to 22.7, 13.04 to 25.56, and 19.62 to 45.35 g m^–2^ min^–1^, respectively. Compared with CK, the average and peak sediment yield rates on alfalfa-covered hillslopes decreased by 12.62% to 85.69% and 11.47% to 56.73%, respectively. These changes in average and peak sediment yield rates indicated that alfalfa coverage altered the sediment generation process to varying extents, with the SS pattern showing the most pronounced reduction. The slope gradient was also a key factor influencing sediment generation. The steeper slope resulted in higher sediment yield rates, with sediment yield rate reaching the peak more quickly.

#### 3.2.3 Infiltration rate

**Figure 5:**
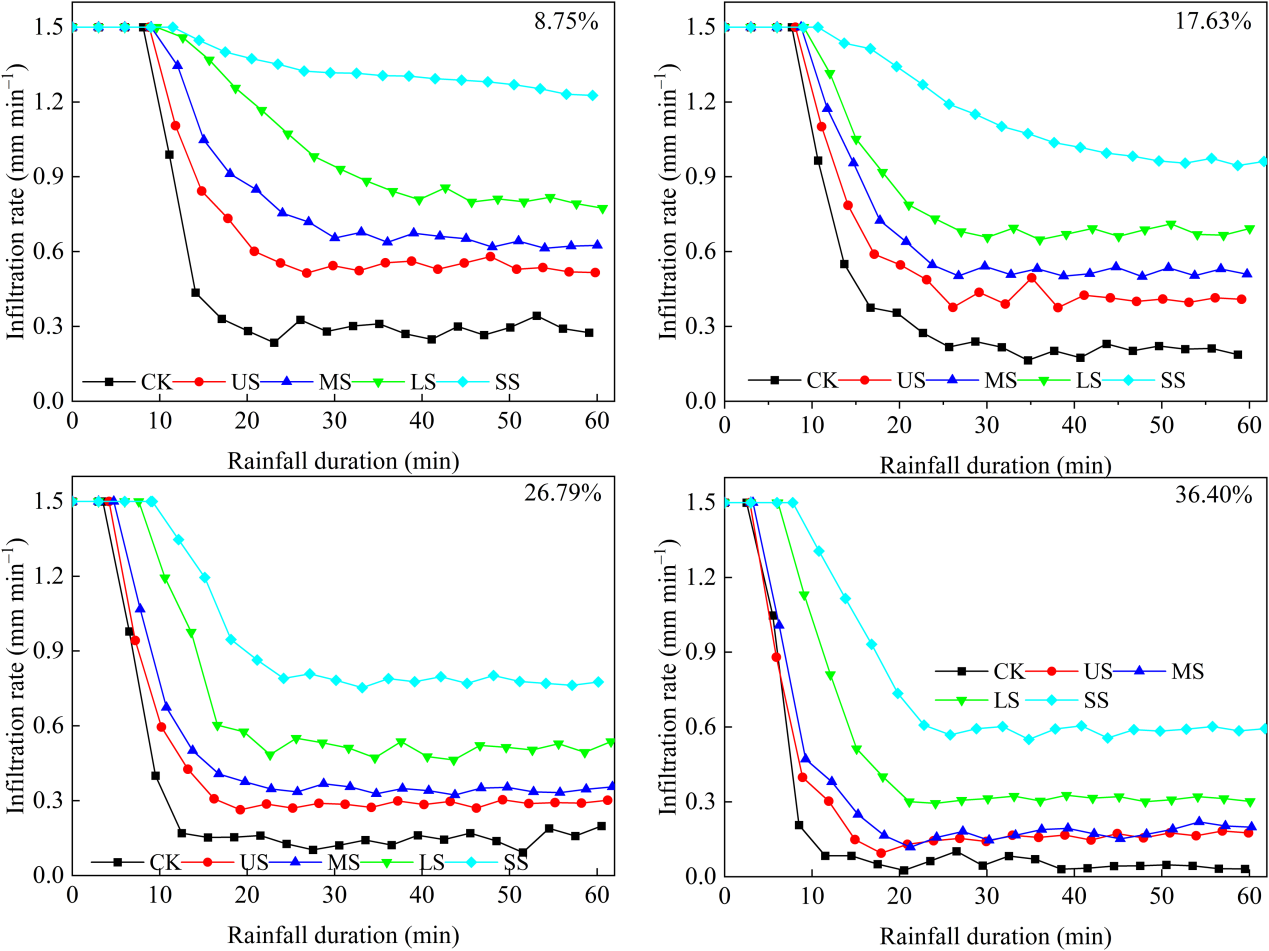
Variation in infiltration rate with rainfall duration and slope gradient of 8.75%, 17.63%, 26.80%, and 36.40%.

**Figure 5** illustrates the variation in infiltration rates with rainfall duration for CK and alfalfa-covered hillslopes. Across CK and alfalfa-covered hillslopes, infiltration rates initially remained high during the early stages and then gradually decreased with rainfall duration, except for the SS with slope gradient of 8.75% and 17.63%, which maintained higher infiltration rates. The average infiltration rates for CK and US, MS, LS, and SS alfalfa-covered hillslopes ranged from 0.17 to 0.50, 0.28 to 0.74, 0.32 to 0.86, 0.32 to 1.05, and 0.79 to 1.35 mm m^-2^ min^-1^, respectively. Compared with CK, the average infiltration rates on US, MS, LS, and SS hillslopes increased by 40.49%–60.00%, 62.53%–83.43%, 93.06–196.67%, and 150.93%–355.86%, respectively, indicating that vegetation cover significantly increased the infiltration rate. On US alfalfa-covered hillslopes, the average infiltration rates at slope gradients of 8.75%, 17.63%, 26.80%, and 36.40% were 0.74, 0.63, 0.42, and 0.28 mm m^-2^ min^-1^, respectively. Overall, the infiltration rates followed the descending order SS, LS, MS, US, and CK and decreased with increasing slope gradient.

### 3.3 The runoff and sediment reduction benefits

**Figure 6:**
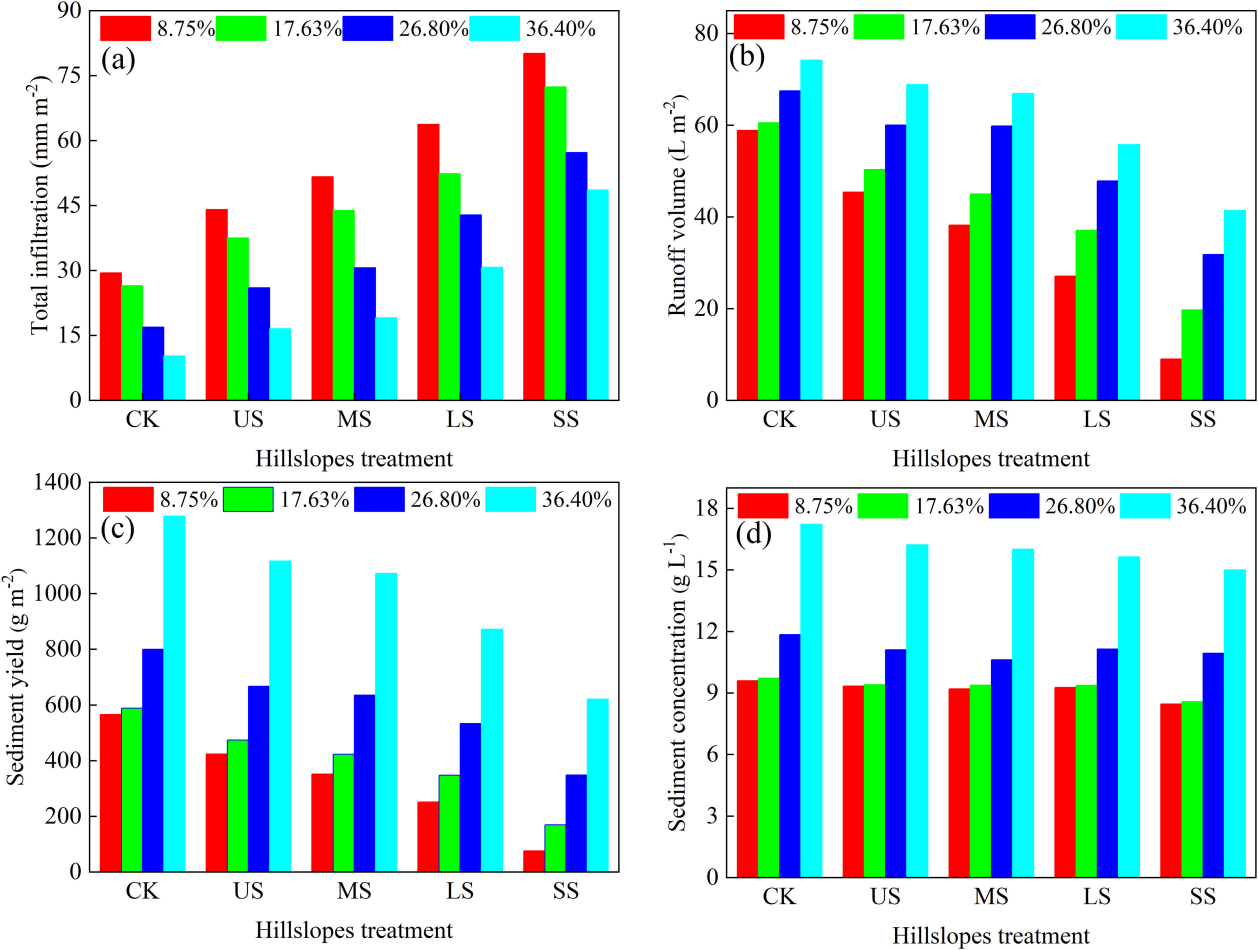
Variation in total infiltration **(a)**, runoff volume **(b)**, sediment yield **(c)**, and sediment concentration **(d)** for different hillslope treatment.

**Figure 6** illustrates the total runoff volume, total sediment yield, total infiltration, and sediment concentration for CK and alfalfa-covered hillslopes. The total runoff volume, total sediment yield, and sediment concentration increased with slope gradient and followed the order CK, US, MS, LS, and SS. However, the total infiltration decreased with slope gradient and followed the order SS, LS, MS, US, and CK. Meanwhile, compared with CK, total runoff, sediment yield, and sediment concentration on alfalfa-covered hillslopes decreased by 5.33 to 49.89 L m^–^², 115.09 to 657.49 g m^–^², and 0.26 to 2.22 g L^–1^, respectively, and total infiltration increased by 6.27 to 50.71 mm^–2^. These findings clearly indicated that alfalfa vegetation significantly enhanced soil infiltration while reducing surface runoff, sediment yield, and sediment concentration. **Table 1** presents the runoff reduction benefit (RRB) and sediment reduction benefit (SRB) of alfalfa-covered hillslopes. Both RRB and SRB decreased with increasing slope gradient and varied with the spatial distribution pattern of vegetation. For alfalfa-covered hillslopes, the RRB with slope gradient of 8.75% was 1.92–3.19 times than that for slope gradient of 36.40%, while the SRB with slope gradient of 8.75% was 1.68–2.34 times than that at 36.40%. Among the different distribution patterns, the SS configuration consistently showed the highest RRB and SRB, whereas the US pattern was relatively poor.

**Table 1:** Runoff and sediment reduction benefits for alfalfa-covered hillslopes at different slope gradients.

	RRB (%)				SRB (%)			
Vegetation spatial distribution patterns	8.75%	17.63%	26.80%	36.40%	8.75%	17.63%	26.80%	36.40%
US	22.91	16.85	11.06	7.19	25.01	19.54	16.60	12.62
MS	35.10	25.61	11.39	9.73	37.77	28.17	20.57	16.14
LS	53.95	38.71	29.12	24.83	55.52	40.92	33.33	31.79
SS	84.67	67.35	52.86	44.20	86.48	71.19	56.46	51.40

RRB, runoff reduction benefits; SRB, sediment reduction benefits.

## 4 Discussion

### 4.1 Effect of alfalfa covers on soil erosion

In the current study, alfalfa-covered hillslopes exhibited good benefits in reducing soil erosion compared with CK. This indicated that plant-covered hillslopes could enhance soil infiltration, thereby reducing runoff volume and concentrated flow scouring, ultimately achieving the objective of mitigating soil erosion (Lamm and Manges, 2000; Levia and Frost, 2003; Van Dijk and Bruijnzeel, 2001). The anti-erosion effect of alfalfa-covered hillslopes was primarily achieved through the canopy, root system, and variation in soil properties (Ghestem et al., 2014; Levia and Frost, 2003; Nardi et al., 2000). The alfalfa canopy decreased the rainfall intensity by redistributing the precipitation to increase stem flow and canopy interception (Van Dijk and Bruijnzeel, 2001), thus reducing the direct raindrops’ impact on the soil surface and minimizing soil aggregation disruption (Hu et al., 2023; Zi et al., 2024). Additionally, the presence of vegetation increased the surface roughness, which further reduced the overland flow velocity and mitigated the soil detachment and sediment transport by flowing water (Li et al., 2024a; Yao et al., 2023b). In this study, compared with CK, the total runoff volume and sediment yield on alfalfa-covered hillslopes decreased by 7.19% to 84.67% and 12.62% to 86.48%, respectively. Zi et al. (2024) reported that the runoff depth and sediment yields for bare soil were 1.19 to 1.64 times and 1.92 to 3.16 times those of hillslopes covered with 50% Nostoc commune, respectively. The difference in benefits in reducing runoff and sediment might be attributed to variations among plant species and herbage spatial distribution patterns (De Baets et al., 2006; Yao et al., 2023a).

The preventive and mitigating efficiency of root systems on soil erosion was primarily manifested through physical consolidation and chemical bonding (Gyssels et al., 2005; Wang and Zhang, 2017; Yao et al., 2023a). The chemical bonding by root systems primarily involved sugars, enzymes, and other organic compounds, which promoted the formation and stabilization of soil organic matter and soil aggregates, thereby enhancing soil stability (Atere et al., 2020; Le Bissonnais et al., 2018; Nardi et al., 2000). Meanwhile, soil particles were adsorbed by the roots, forming root–soil composites and enhancing soil erosion resistance (Entezami et al., 2024; Ghestem et al., 2014; Yang et al., 2024; Zhao et al., 2024). Additionally, the roots physically intertwined and compressed the soil, forming a network structure that improved soil stability, shear strength, and infiltration capacity (Song et al., 2025; Wang et al., 2022a, 2024a). In this study, compared with CK, the infiltration on alfalfa-covered hillslopes increased by 40.49% to 355.86%, demonstrating the role of root systems in improving soil permeability and infiltration. The finding of Liu et al. (2022b) indicated that the presence of *Elymus tangutorum* clearly increased the rainfall infiltration rate by 73% compared with CK. The difference in rainfall infiltration for alfalfa and *Elymus tangutorum* might be induced by different root types (De Baets et al., 2006; Entezami et al., 2024; Futerman et al., 2025; Gyssels et al., 2005). In general, the taproot system and the fibrous root system exhibited different soil anti-erosion functions (Le Bissonnais et al., 2018; Wang and Zhang, 2017).

### 4.2 Fluctuation variation in the soil erosion process

#### 4.2.1 Infiltration rate

In the current study, the infiltration rate showed a downward trend with rainfall duration, and the infiltration rate for alfalfa-covered hillslopes was obviously higher than that for CK (**Figure 5**). This pattern might be attributed to the initially low soil moisture content and the relatively weak root water absorption capacity during the early rainfall period, while soil infiltration was primarily governed by initial moisture in the early rainfall period (Han et al., 2024; Lin et al., 2024; Niu et al., 2024; Zi et al., 2024). Consequently, the soil infiltration rate was relatively high, and differences between treatments were minimal. As the rainfall continued, the soil moisture content gradually increased and the roots began to absorb a large amount of water, making the infiltration rate for alfalfa-covered hillslopes higher than that for CK (Li et al., 2024b; Niu et al., 2024; Wang et al., 2023b). Meanwhile, the large amount of water was absorbed by the roots, and the water content in the soil pores rise rapidly, resulting in an increase in the proportion of soil pores filled with water, narrowing of the water flow channels, and an increase in infiltration resistance (Hu et al., 2023; Wang et al., 2022a, 2023b).

#### 4.2.2 Runoff rate

In the current study, the runoff rate generally showed an increasing trend firstly and then fluctuated stably with rainfall duration extension. During the rainfall process, the runoff volume was determined to some extent on the dynamic balance between precipitation and infiltration rate (Bahddou et al., 2023; Leung et al., 2017). At the early period of rainfall, the soil infiltration rate was higher, resulting in a lower runoff rate (Lin et al., 2024; Vinatier et al., 2024; Wang et al., 2023a; Wu et al., 2023). As rainfall continued, the soil gradually became saturated and soil crust was formed on the surface, which led to an increased conversion of precipitation into runoff and a corresponding rise in runoff volume (Bahddou et al., 2023; Chen et al., 2024a; Liu et al., 2022b). When the precipitation and soil infiltration reached a dynamic balance, the runoff generation process tended to be stable (Wang et al., 2022b, 2023a). The variation coefficients of runoff rates across the entire rainfall process are illustrated in **Table 2**. Overall, the variation coefficients for CK were lower than that for alfalfa-covered hillslopes, indicating a lower degree of runoff variability for CK. This difference was likely due to the presence of vegetation, which altered the runoff generation process (Futerman et al., 2025; Leung et al., 2017; Van Dijk and Bruijnzeel, 2001). There were certain shielding and aggregation effects of vegetation cover on rainfall, resulting in a certain delay effect on runoff (Chen et al., 2024a; Futerman et al., 2025; Oliveira et al., 2024; Zhang et al., 2024b). The aggregation effect of vegetation on rainfall broke the dynamic balance between soil infiltration and rainfall and caused certain fluctuations in runoff (Li et al., 2024b; Yubonchit et al., 2025).

**Table 2:** Variation coefficients of runoff rate across the entire rainfall process for CK and alfalfa-covered hillslopes.

Slope gradient	Vegetation spatial distribution patterns				
	CK	US	MS	LS	SS
8.75%	0.29	0.31	0.36	0.49	0.41
17.63%	0.30	0.31	0.33	0.35	0.50
26.80%	0.28	0.28	0.29	0.32	0.36
36.40%	0.29	0.27	0.29	0.32	0.36

CK, bare soil; US, upper hillslope planting; MS, middle hillslope planting; LS, downstream hillslopes planting; SS, equally spaced planting.

#### 4.2.3 Sediment yield rate

**Figure 7:**
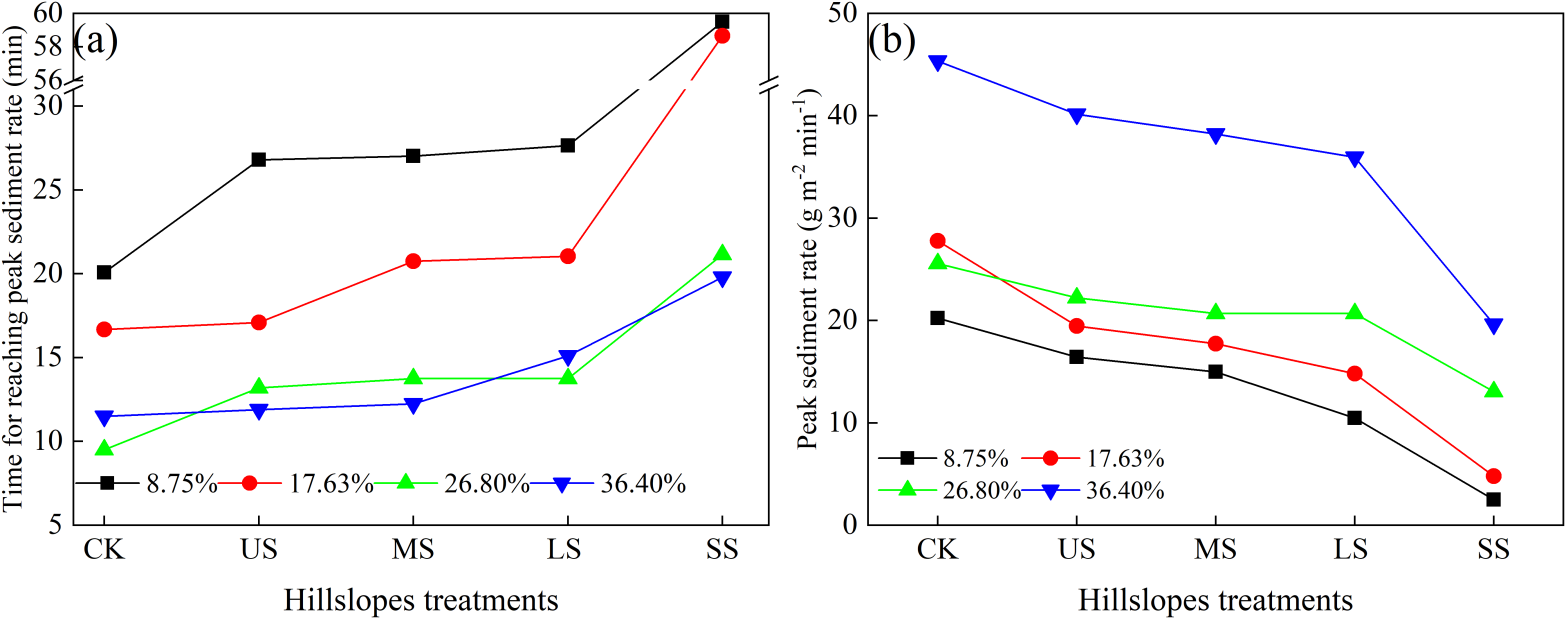
Variations in time to reaching peak sediment yield rate **(a)** and peak sediment yield rate **(b)** for different hillslope treatment.

For alfalfa-covered and CK hillslopes, the sediment yield rate increased firstly and then decreased. Sediment sources on the hillslopes were composed of particles separated by raindrop splash and scouring by runoff (Wang et al., 2024c; Zhang et al., 2003). At the early period of rainfall, there were soil particles separated by raindrop splash, providing loose sediment for the runoff to carry (Li et al., 2024c; Lv et al., 2023; Wang et al., 2023c). However, due to the limited runoff during the initial phase, the sediment transport capacity was low, resulting in a relatively small sediment yield rate (Huang et al., 2021; Polyakov and Nearing, 2003; Wang et al., 2015). As rainfall persisted, both the runoff volume and its sediment transport capacity increased, leading to a rise in the sediment yield rate (Ali et al., 2013; Mu et al., 2019; Polyakov and Nearing, 2003; Zambon et al., 2021). The increasing runoff depth reduced the kinetic energy of the raindrops’ impact, and scouring by flowing water was a main eroding force. Meanwhile, the soil surface pores were gradually filled with fine particles, forming a dense soil crust and leading to an increase in soil stability and anti-erosion ability (Liu et al., 2024, 2022a). Once the overland flow stabilized, sediment separated by runoff diminished, leading to a reduction in the sediment yield rate. As shown in **Figure 7a**, the peak sediment yield rate occurred earlier on CK hillslopes than that on alfalfa-covered hillslopes. This was likely because soil crusts and steady overland flow developed more rapidly for CK (Chen et al., 2022; Dan et al., 2024; Oliveira et al., 2024). Meanwhile, the time to reach the peak sediment yield rate on alfalfa-covered hillslopes followed the order SS, LS, MS, and US (**Figure 7a**). The peak sediment yield rate followed the order CK, US, MS, LS, and SS (**Figure 7b**).

### 4.3 Difference in soil erosion for different alfalfa spatial distributions

The results indicated that total runoff and total sediment followed the order US, MS, LS, and SS, underscoring the complexity of vegetation spatial distribution in affecting the hillslope erosion dynamics (Feng et al., 2018; Hu et al., 2023). Vegetation located upstream of hillslopes (US) implied that the downstream areas lacked a direct shielding effect from vegetation, thus increasing the susceptibility of soil detachment and sediment transport by runoff (Li et al., 2024b; Niu et al., 2024). The absence of vegetation in downstream areas allowed the sediment to flow unobstructed downslope, increasing the concentrated flow erosion (Hu et al., 2023; Li et al., 2024b). In contrast, vegetation located downstream of hillslopes effectively intercepted sediments from upstream, reduced its downward transport, and facilitated sediment deposition via its dense root network and surface cover, forming a natural deposition zone (Futerman et al., 2025; Lin et al., 2024). This “sediment dam” effect greatly alleviated erosion in downstream areas, highlighting the critical role of downstream vegetation in mitigating erosion (Mu et al., 2019; Vinatier et al., 2024). Vegetation patterns located mid-hillslopes (MS) exhibited runoff and sediment reduction effects that lie between those for US and LS, which reduced the erosive force by reducing the velocity of overland flow and enhancing the hillslope surface roughness (Bahddou et al., 2023; Hu et al., 2023). Uniformly distributed vegetation enhanced the canopy interception, facilitated the runoff dispersion, and reduced the runoff velocity, thereby minimizing concentrated flow erosion (Bahddou et al., 2023; Oliveira et al., 2024). Additionally, the extensive distribution of root network formed a multi-layered erosion protection system (Entezami et al., 2024; Song et al., 2025). Thus, the SS pattern provided optimal erosion mitigation by changing runoff paths, improving soil stability, and reducing erosive energy.

### 4.4 Significance and limitation of this study

This study investigated the effect of herbaceous vegetation spatial distribution patterns on hillslope erosion process with different slope gradients (8.75%–36.40%) and a rainfall intensity of 90 mm h^–1^ on the Loess Plateau. The results indicated that alfalfa-covered hillslopes altered the soil erosion process by delaying the initial runoff generation time and reducing the runoff rate and sediment yield rate. The findings also highlighted the influence of vegetation spatial distribution patterns on hillslope erosion ranging from gentle to steep. The study enhanced our understanding on how herbaceous vegetation and its distribution patterns affected soil erosion and provided more scientific guidance to innovate soil and water conservation regulations on the Loess Plateau. Despite the significant results, the current study only validated the impact of a single herbaceous vegetation spatial distribution pattern on soil erosion. The effect of composite vegetation on soil erosion required additional verification. Moreover, the applicability of these findings needs to be validated in other erosion-prone regions.

## 5 Conclusion

The current study investigated the impacts of alfalfa spatial distribution pattern on hillslope erosion process. The results indicated that the initial runoff generation time on alfalfa-covered hillslopes was delayed by 2.50 to 11.50 min in comparison with CK. The initial runoff generation time for all treatments was advanced with increasing slope gradient. The average runoff rates for CK and alfalfa-covered hillslopes were 0.18 to 1.24 L m^–2^ min^–1^, respectively. Compared with CK, the average runoff rates for alfalfa-covered hillslopes decreased by 7.18% to 83.77%. The average sediment yield rates for CK and alfalfa-covered hillslopes were 1.50 to 21.32 g m^–2^ min^–1^, and the peak sediment yield rates were 10.74 to 45.35 g m^–2^ min^–1^. Compared with CK, the average and the peak sediment yield rates for alfalfa-covered hillslopes decreased by 12.62% to 85.69% and 11.47% to 56.73%, respectively. The average infiltration rates for CK and alfalfa-covered hillslopes with slope gradient of 8.75%–36.40% were 0.17 to 0.50 and 0.28 to 1.35 mm m^-2^ min^-1^, respectively. Meanwhile, slope gradient is an important factor affecting the runoff and sediment generation. As the slope gradient increased, the RRB and SRB on alfalfa-covered hillslopes gradually decreased, and there were obvious differences in RRB and SRB on alfalfa-covered hillslopes with different vegetation spatial distribution patterns. Due to the shielding and protection effects of alfalfa, initial runoff generation time was delayed, and the runoff and sediment yield rates were reduced. The alfalfa-covered hillslopes yielded good soil and water conservation benefits. The results from the current study provided scientific reference to understand the effect of herbaceous spatial distribution pattern on anti-erosion.

## Data Availability

The original contributions presented in the study are included in the article/supplementary material. Further inquiries can be directed to the corresponding authors.
